# Fecal carriage and genetic characteristics of carbapenem-resistant enterobacterales among adults from four provinces of China

**DOI:** 10.3389/fepid.2023.1304324

**Published:** 2024-01-03

**Authors:** Yuanyuan Li, Lan Ma, Xinying Ding, Rong Zhang

**Affiliations:** ^1^ Department of Clinical Laboratory, The First Affiliated Hospital of Henan University of Science and Technology, Luoyang, Henan, China; ^2^ Department of Clinical Laboratory, Second Affiliated Hospital of Lanzhou University, Lanzhou, Gansu, China; ^3^ Department of Clinical Laboratory, Zibo First Hospital, Zibo, Shandong, China; ^4^ Department of Clinical Laboratory, Second Affiliated Hospital of Zhejiang University, Hangzhou, Zhejiang, China

**Keywords:** carbapenem-resistant enterobacterales, *bla_NDM_*, carbapenemase genes, fecal carriage, dissemination

## Abstract

Carbapenem-resistant *Enterobacterales* (CRE) is a global concern. This study investigated the prevalence of fecal colonization carriage and clonal dissemination of CRE among population in four provinces of China. A total of 685 stool samples were collected from four provinces in China. Among these samples, 141 and 544 were obtained from healthy and hospitalized individuals, respectively. The overall fecal carriage rate was 9.6% (65/685) with 4.26% (95% CI: 0.9–7.6) in healthy individuals and 10.84% (95% CI: 8.2–13.5) in hospitalized patients. The highest prevalence was in Henan province (18.35%, 95% CI: 9%–18.7%). Sixty-six CRE isolates were identified in *Escherichia coli* (56.06%, 37/66), *Klebsiella* (15.15%, 10/66), *Citrobacter* (13.63%, 9/66), *Enterobacter* (12.12%, 8/66), and *Atlantibacter* (1.51%, 1/66). All CRE strains carried carbapenemase genes and multiple antibiotics resistance genes, *bla_NDM−5_* (77.27%, 51/66) was the most common carbapenemase gene, followed by *bla_NDM−1_* (19.69%, 13/66). Antibiotic resistance genes, including *bla_IMP−4_,* and the colistin colistin resistance (mcr-1) gene were also identified. All CRE isolates belonged to different sequence types (STs). ST206 (36.84%, 14/38) in *E. coli* and ST2270 (60%, 6/10) in *Klebsiella* were significantly dominant clones. The results indicated the prevalence of CRE fecal carriage among adults of China, mostly *bla_NDM_*-producing *E coli*, which pose significant challenges for clinical management. Screening for CRE colonization is necessary to control infection.

## 1. Introduction

Carbapenem-resistant *Enterobacterales* (CRE) is experiencing rapid global dissemination. According to data from the China Antimicrobial Surveillance Network (CHINET), the rates of carbapenem resistance in *Escherichia coli* and *Klebsiella pneumoniae* in China increased from 0% and 2.9% in 2005 to 2% and 21.4% in 2022, respectively. In Japan, the yearly number of reported cases of CRE infections has remained rather stable since 2015, with a stool CRE carriage rate of 12.2% (1). However, this rate drastically increased in 2018. In Taiwan, the prevalence rates of CRE among all clinical Enterobacteriaceae isolates have risen to approximately 7% in 2013; 2017 data from in intensive care units (ICUs) revealed a prevalence rate of 15.3% (2). Because of the increasing prevalence and limitation of treatment options, CRE infection leads to higher resistance rate and a considerable mortality risk (3).

Carbapenemases consist of five major enzymes, including *K. pneumoniae* carbapenemases (KPC), imipenemase metallo-β-lactamase (IMP), veronaintegron-encoded metallo-β-lactamase (VIM), New Delhi metallo-β-lactamase (NDM), and oxacillinase-48-type carbapenemases (OXA-48) (4). The production of these enzymes constitutes the primary resistance mechanism and is found in approximately 85% of CRE cases worldwide (5). Carbapenemase-encoding genes are usually present on mobile genetic elements, which are driving the increasing rapid spread and extensive distribution of CRE through multiple routes (6, 7). Therefore, the infection control risk of CRE is likely higher (8). CRE colonization is significantly associated with CRE infection.

Recently, rectal or perirectal swabs have been utilized for screening based on Centers for Disease Control and Prevention and Healthcare Infection Control Practices Advisory Committee recommendations. These swabs might produce higher yields compared to testing of other body sites (9). Early identification of CRE and detection of asymptomatic carriers through rectal surveillance is exceedingly important and can reduce dissemination (10, 11). This fecal carriage is an important concern for the infection control practitioner because the gut can be considered a reservoir for exchanging resistance genes among bacteria. As a strategy to control cross-transmission, screening of CRE intestinal carriage is used by many hospitals, especially in ICUs. A previous study reported CRE carriage rates of 0.5%–12.2% among hospital patients in different areas of the world (12–16), with rates of 75.5% in patients with hematological malignancies (17) and 6.79% in those with liver disease (18).

However, minimal data regarding the prevalence and mobile resistance elements of CRE fecal carriage colonization among population with various regions are available. The dearth of reliable information on the CRE-mediated infection impacts patient health. Increased knowledge would aid the understanding of CRE transmission characteristics. Therefore, addressing the prevalence and molecular epidemiological of CRE requires further attention.

In this study, we isolated and analyzed stool samples in various regions in China, including Gansu, Zhejiang, Henan, and Shandong provinces. The data provided up-to-data knowledge on epidemiological and genomic levels of CRE fecal carriage.

## 2. Materials and methods

### 2.1. Retrospective screening of CRE isolates

A total of 685 fecal individual samples were collected from five hospitals in four distinct provinces. Convenient sampling was used. Healthy individual stool samples were also collected. No duplicate samples were isolated from the same individual. The number of specimens for each region was as follows: Zhejiang (*n* = 274), Henan (*n* = 195), Shandong (*n* = 116), and Gansu (*n* = 100). Among the 685 fecal swabs, 141 samples were obtained from healthy individuals. All strains were identified by matrix-assisted laser desorption/ionization- time-of-flight mass spectrometry (MALDI-TOF MS) using a Microflex LT instrument (Bruker Daltonik GmbH, Bremen, Germany).

### 2.2. Detection of carbapenem genes

All CRE were isolated according to a previously described method (19, 20). Briefly, approximately five grams of each fresh fecal specimen was directly cultured in 5 ml of LB broth (Luqiao, Beijing, China) and enriched at 35°C for 22–24 h. Ten microliter aliquots of the culture were inoculated onto two selective China Blue agar plates to screen for non-susceptible *Enterobacterales* isolates. One contained 0.3 μg/ml meropenem and the other 8 μg/ml Ceftazidime-avibactam. The plates were incubated at 35°C for 16–18 h. Colonies on the plate were selected for further purification and confirmation as *Enterobacterales*. Isolates from the same patient exhibiting different colonial morphotypes were also studied. Only one suspected colony was analyzed per sample. Colonies were screened for the presence of five common carbapenemase genes (*bla*_KPC_, *bla*_NDM_, *bla*_OXA−48−like_, *bla*_IMP_, and *bla*_VIM_) using the NG-Test Carba5 assay (NG Biotech, Guipry, France) as previously described (21). According to the manufacturer's instructions, a colony of a pure cultivated strain was mixed with five drops of lysis buffer. After vortexing, the mixture was left at 20–25°C for 10 min. Once hundred microliters of the mixture was transferred to the NG-Test Carba5 cassette. The results were read after 15 min of incubation.

### 2.3. Antimicrobial susceptibility testing

Susceptibility to antimicrobials was tested by the broth microdilution method. The tested antimicrobials included amikacin, imipenem, meropenem, ertapenem, cefmetazole, cefotaxime, aztreonam, ceftazidime, tazobactam and piperacillin, cefepime, ciprofloxacin, colistin, tigecycline, ceftazidime-avibactam, and cefoperazone-sulbactam. Interpretation of minimum inhibitory concentration (MIC) breakpoints was based on guidelines given by the CLSI criteria (22). European Committee on Antimicrobial Susceptibility Testing breakpoints were used for tigecycline (23).

### 2.4. Whole genome sequencing and bioinformatics analysis

Total genomic DNA was extracted from overnight cultures of 66 carbapenem-resistant Enterobacterales isolates using the PureLink Genomic DNA Mini Kit (Invitrogen, Carlsbad, CA, USA) according to the provided instructions. All isolates harboring carbapenem genes underwent whole genome sequencing using the HiSeq platform (Illumina, San Diego, CA, USA). De novo assembly was performed using SPAdes version 3.15.1 (24). The locations of carbapenem genes were determined by NCBI databases. Multilocus sequence typing (MLST) of strains was performed using the MLST tool (https://github.com/tseemann/mlst) and contigs were assembled. Sequence types (STs) and corresponding MLST gene allele profiles were recorded in BioNumerics v7.6.3 (Applied Maths, Sint-Martens-Latem, Belgium). The assembled sequences were analyzed using the ResFinder bioinformatic database (25) and ISFinder (https://www-is.biotoul.fr/) bioinformatic tools. Advanced Heatmap Plots for antimicrobial resistance (AMR) genes were performed using the OmicStudio tools (https://www.omicstudio.cn). The molecular features of each strain were visualized using the iTOL online tool (26) and Phyloviz (http://www.phyloviz.net/) for eBURST analysis.

Accession numbers

All sequences have been deposited at GenBank under BioProject number PRJNA1023209.

### 2.5. Statistical analysis

Descriptive statistics were used to summarize the epidemiologic characteristics of CRE strains. For categorical variables, the percentage of CRE strains in each category was calculated. All analyses were performed using the SPSS Statistics (version 21; IBM, Armonk, NY, USA).

## 3. Results

### 3.1. Prevalence characterization of CRE strains in four provinces in China

Fecal colonization rates of CRE in samples collected in four different provinces were determined. The largest number of samples were from the provinces of Zhejiang (*n* = 274) and Henan (*n* = 195). Of the 685 human fecal samples, 65 tested positive for CRE. Sixty-six CRE strains were isolated from 65 patient, representing a carriage rate of 9.6% (95% CI: 7.4%–11.9%).

A total of 141 fecal samples were obtained from healthy persons. From these samples, six (4.26%, 95% CI: 0.9–7.6) CRE strains were identified, which were isolated from three males and three females with a median age (IQR) of 58.5 (54.25–65) years. Five strains were isolated from 100 samples (5%, 95% CI: 0.7%–9.3%) from Zhejiang and 1 strain was isolated from 41 samples (2.44%, 95% CI: 2.5%–7.4%) from Henan.

A total of 544 fecal samples were gathered from hospitalized patients, from which 59 (10.84%, 95% CI: 8.2%–13.5%) CRE strains were isolated. The prevalence of CRE in the hospitalized patients was significantly higher than the healthy individuals (*P* = 0.001). These patients included 35 males and 24 females with a median age (IQR) of 57 (48–67) years. The prevalence rate was highest in Henan province (16.23%, 95% CI: 10.3–22.1%), followed by Zhejiang province (14.37%, 95% CI: 9.1%–19.6%). Seven samples tested positive in Gansu province, yielding a prevalence rate of 7% (95% CI: 1.9%–12.1%). Only two samples from Shandong province were positive, indicating a prevalence rate of 1.72% (95% CI: 0.7%–4.1%) (Table 1).

**Table 1:** Prevalence of CRE fecal carriage from hospitalized inpatients and healthy individuals.

Types of samples	characteristics of samples	No. samples	No. of positive	Type of carbapnemase (*n*)	Prevalence% (95% CI) or *n* (%) or median (IQR)
Healthy individuals	Zhejiang	100	5	*bla_NDM_* (5)	5 (0.7–9.3)
	Henan	41	1	*bla_NDM_* (1)	2.44 (2.5–7.4)
		141	6		4.26 (0.9–7.6)
	Male	79	3		3.79
	Female	62	3		4.83
Hospitalized patients	Zhejiang	174	25	*bla_NDM_* (25)	14.37 (9.1–19.6)
	Henan	154	25	*bla_NDM_* (23)	16.23 (10.3–22.1)
				*bla_NDM _+ bla_IMP_*(1)	
				*bla_IMP_*(1)	
	Gansu	100	7	*bla_NDM_* (7)	7 (1.9–12.1)
	Shandong	116	2	*bla_NDM_* (2)	1.72 (0.7–4.1)
		544	59		10.84 (8.2–13.5)
	Male	273	35		12.82
	Female	271	24		8.85
Total		685	65		9.6 (7.4–11.9)

### 3.2. Antibiotic resistance patterns of CRE strains

Antimicrobial susceptibility testing was performed on all carbapenem-resistant strains using 14 clinical antibiotics. The CRE strains exhibited 100% resistance to multiple antibiotics that included ceftazidime, cefotaxime, ertapenem, and cefoperazone-sulbactam. Additionally, notable high resistance rates were evident for imipenem (98.53%), meropenem (94.12%), ceftazidime-avibactam (95.59%), piperacillin-tazobactam (91.18%), and cefepime (89.71%). Low resistance rates were observed for polymyxin B (1.47%), tigecycline (1.47%), and amikacin (1.47%). A comprehensive overview of resistance profiles of the 66 CRE positive strains against 14 antimicrobial agents is presented in Table 2. *E. coli* strains displayed the same resistance to antibiotics agents.

**Table 2:** Antimicrobial susceptibility testing results among all CRE samples and CREC.

Antimicrobial	All strains (*n* = 66)				*E. coli* (*n* = 37)			
	R%	S%	MIC50 (ug/ml)	MIC90 (ug/ml)	R%	S%	MIC50 (ug/ml)	MIC90 (ug/ml)
Imipenem	98.53	0	8	32	98.53	0	8	32
Meropenem	94.12	1.47	16	64	94.21	1.47	16	64
Ceftazidime	100	0	>128	>128	100	0	>128	>128
Cefotaxime	100	0	>128	>128	100	0	>128	>128
Ertapenem	100	0	32	64	100	0	32	64
Ceftazidime-avibactam	95.59	4.41	>64/4	>64/4	95.59	4.41	>64/4	>64/4
Piperacillin -Tazobactam	91.18	1.47	256/4	>256/4	91.18	1.47	256/4	>256/4
Polymyxin B	1.47	98.53	1	2	1.47	98.53	1	2
Tigecycline	1.47	98.53	≤0.25	≤0.25	1.47	98.53	≤0.25	≤0.25
Ciprofloxacin	60.29	38.24	4	>32	60.29	38.24	4	>32
Cefepime	89.71	0	32	>64	89.71	0	32	>64
Amikacin	1.47	98.53	≤4	≤4	1.47	98.53	≤4	≤4
Aztreonam	19.12	73.53	≤4	32	19.12	73.53	≤4	32
Cefoperazone -Sulbactam	100	0	256/4	>256/4	100	0	256/4	>256/4

### 3.3. Diversity of carbapenem resistance genes among CRE

**Figure 1:**
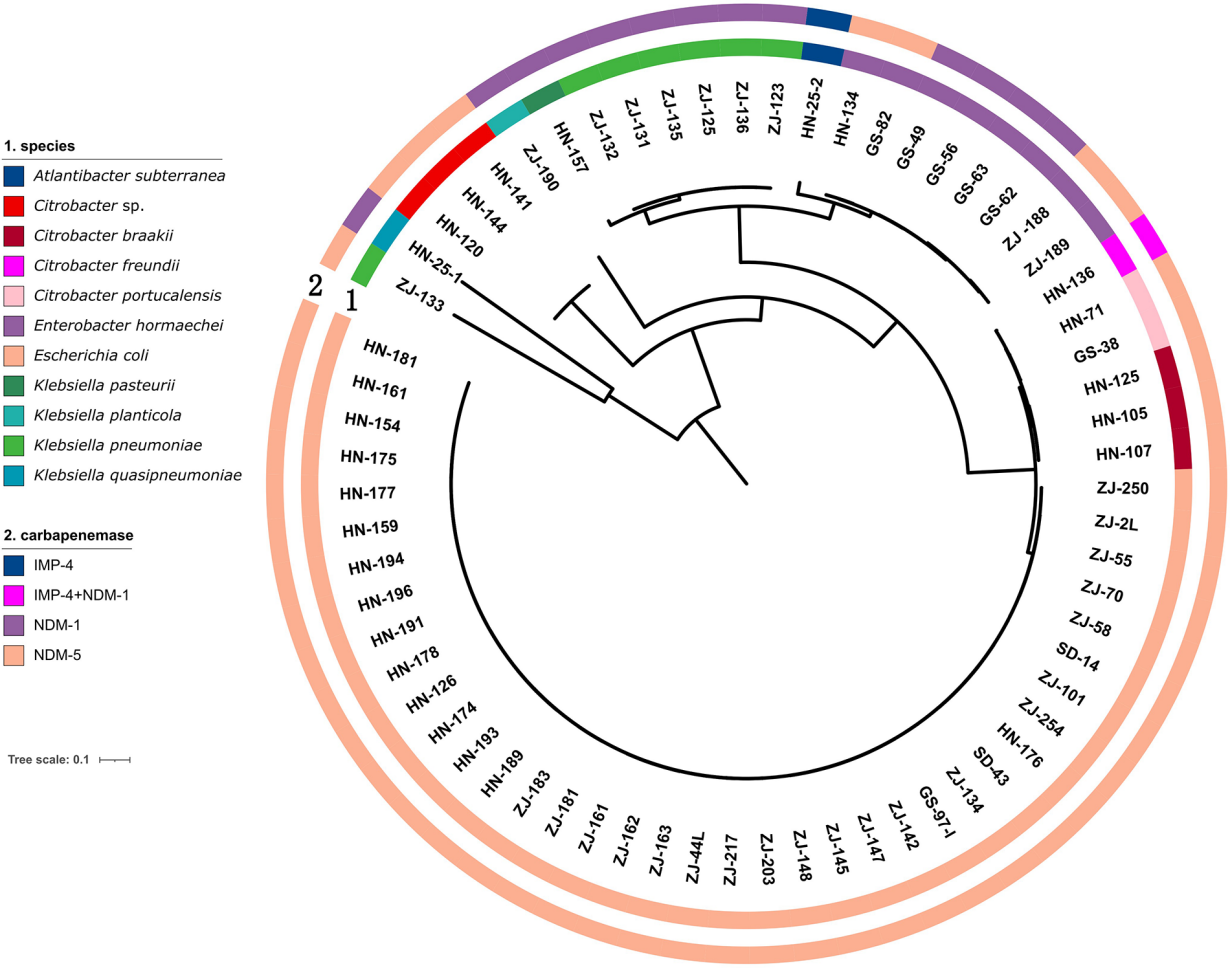
Genetic analysis of 66 CRE isolates. Circles 1 and 2 indicate the isolates and the carbapenemase genes, respectively.

Whole genome sequencing analysis revealed that the CRE comprised different genera. *Escherichia* was the most prevalent genus (57.57%, 38/66), followed by *Klebsiella* (15.15%, 10/66), *Citrobacter* (13.63%, 9/66), *Enterobacter* (12.12%, 8/66), and *Atlantibacter (*1.51%, 1/66). All *Escherichia* and *Enterobacter* were identified as *E*. *coli* and *E. hormaechei*, respectively. In contrast, *Klebsiella* consisted of various species, including seven *K. pneumoniae* isolates, one *K*. *quasipneumoniae*, one *K*. *pasteurii,* and one *K*. *planticola*. *Citrobacter* isolates included three *C. braakii*, two *C. portucalensis*, one *C. freundii* and three other *Citrobacter* isolates (Figure 1).

**Figure 2:**
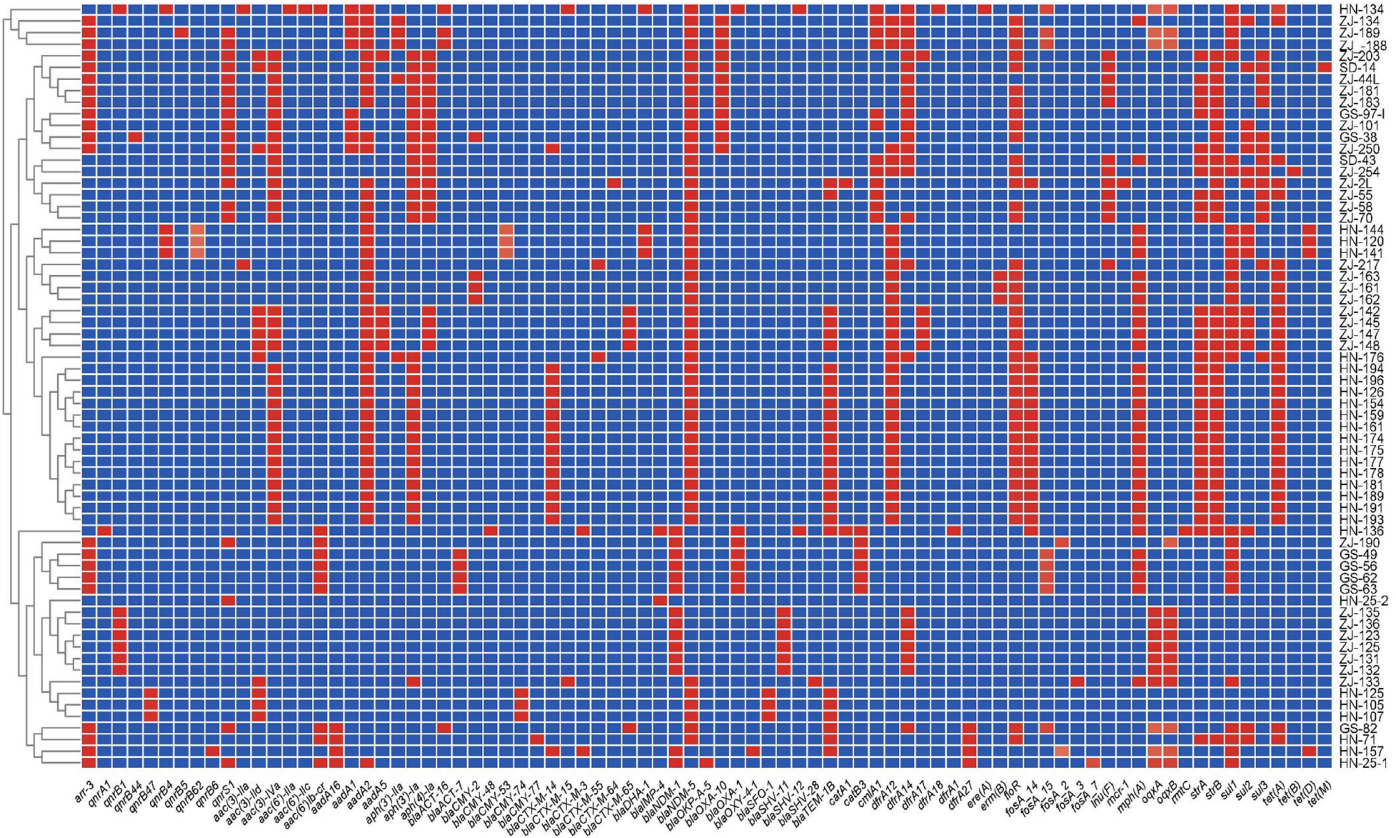
Heatmap of antimicrobial resistance genes of 66 CRE strains collected in the four provinces in China. The *X*-axis denotes the antimicrobial resistance genes harbored by each strain. The *Y*-axis denotes the CRE strains. Labels in the *Y*-axis represent the source of the strain. ZJ, Zhejiang; GS, Gansu; HN, Henan; and SD, Shandong. Red and blue colors indicate the presence and absence of the corresponding antimicrobial resistance genes, respectively.

All CRE isolates encoded carbapenemase genes (Figure 2). Among 66 CRE isolates, 77.27% (51/66) harbored *bla_NDM−5_* and 19.69% (13/66) harbored *bla_NDM−1_*. Both *bla_IMP−4_* and *bla_NDM−1_* were detected in one *C. freundii* isolated from a male, 59-year-old patient with a tumor. The patient had no history of travel in the preceding 6 months. The *bla_IMP−4_* gene was detected in one *Atlantibacter subterranean* isolated from Henan. One *E. coli* strain harbored both *bla_NDM−5_* and *mcr-1* gene; it exhibited resistance to colistin. The prevalence of the *bla_NDM_* genes varied among different strains of CRE. Specifically, the *bla_NDM−5_* gene was detectable in all tested *E. coli* isolates, while it was present in only 10% of *Klebsiella* isolates. *bla_NDM−1_* was detected in all *Enterobacter* isolates and 90% of *Klebsiella* isolates.

Sequence analysis identified various acquired drug resistance genes among the 66 CRE isolates (Figure 2). A number of diverse acquired resistance genes were identified, including aminoglycosides resistance genes (*aac(3)-IIa*, *aac(3)-IId*, *aac(3)-IV*, *aac(6')-Ib-cr*, *aac(6')-IIa*, *aac(6’)-IIc*, *aadA16*, *aadA2*, *aadA5*, *aph(3')-Ia*, *aph(3’)-IIa*, and *aph(4)-Ia*), quinolone resistance genes (*qnrS1*, *qnrA1*, *qnrB4*, *qnrB44*, *qnrB47*, *qnrB5*, *qnrB62*, *qnrB6*, and *qnrS1*), extended spectrum beta-lactamase (ESBL) resistance genes [*CTX-M-3*, *SHV-12*, *CTX-M-14*, *OXY-4-1*, *SFO-1*, *CTX-M-65*, *CTX-M-15*, tetracycline resistance genes (*tetA*, *tetD*, *tetB*, and *tetM*), *AmpC* β-lactamase gene(*bla*_CMY−48_, *bla*_ACT−17_, *bla*_CMY−157_, *bla*_CMY−82_, *bla*_ACT−25_, *bla*_CMY−2_, *bla*_DHA−1_], and other narrow-spectrum β-lactam resistance genes (*bla*_OXA−1_, *bla*_TEM−1D_, *bla*_OXA−10_, and *bla*_OKP−A−5_, *bla*_SHV−11_, *bla*_SHV−28_). In the 66 CRE isolates, aminoglycoside genes were identified in 58 strains, the most prevalent *aac(3)-Iv* (*n* = 33). Thirty-seven CRE isolates also carried AmpC β-lactamase genes, including *bla*_CMY_ (*n* = 14), *bla*_ACT_ (*n* = 8), *bla*_DHA−1_ (*n* = 4). Thirty-seven isolates harbored ESBL genes, including *bla*_CTX−M_ (*n* = 27), *bla*_SFO−1_ (*n* = 3), and *bla*_SHV−12_ (*n* = 2). Finally, 38 strains harbored tetracycline resistance genes, mostly *tetA* (*n* = 32).

### 3.4. Distribution of STs among carbapenemase-producing CRE isolates

**Figure 3:**
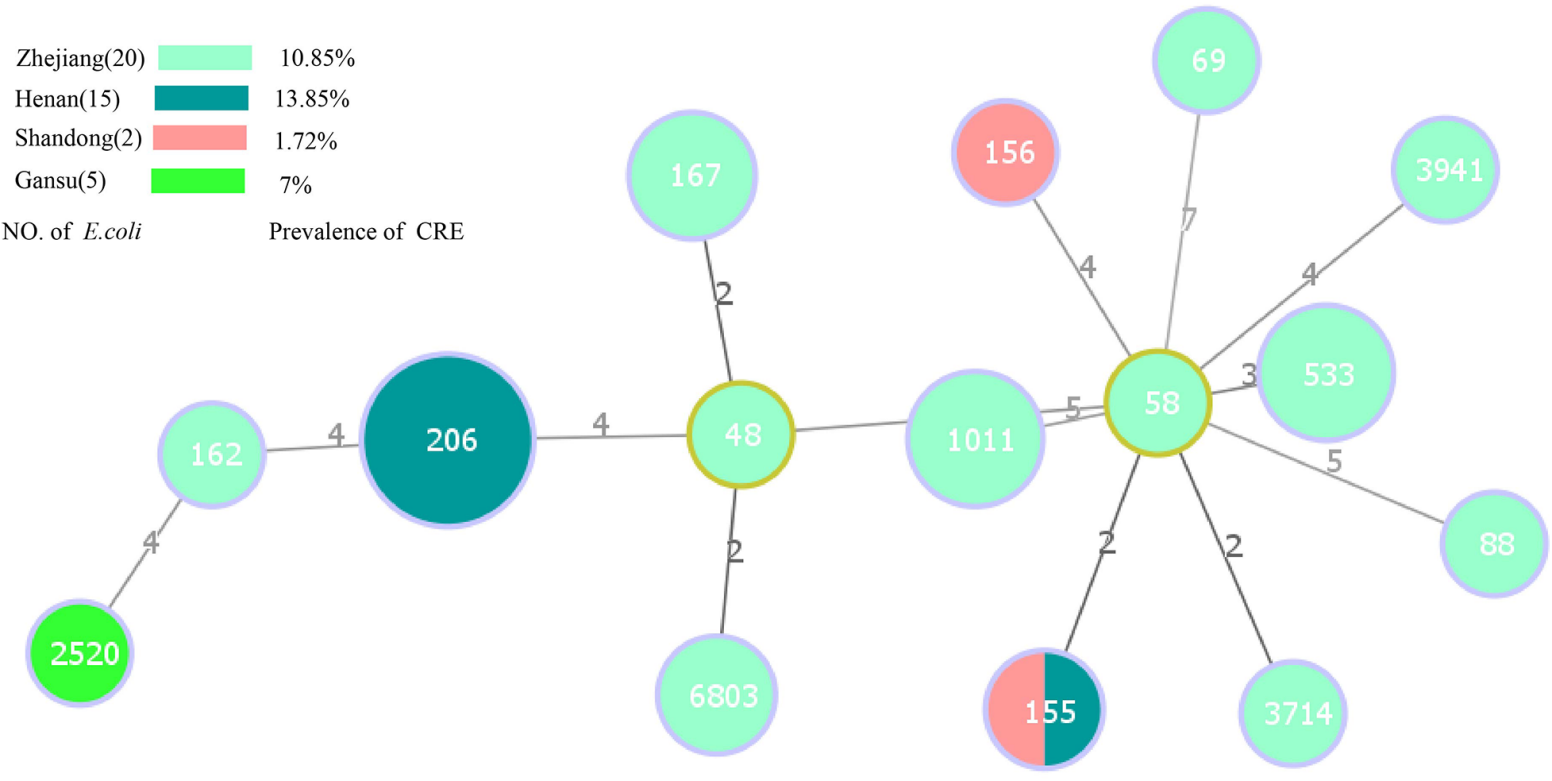
Minimal spanning tree based on multilocus sequence typing of carbapenem-resistant *E. coli*. Colored circles represent different location. The circle sizes are proportional to the number of STs. The length of the connecting lines represents the number of target genes with different alleles. Red dots indicate ST48 and ST1011 *E. coli* strains isolated from healthy individuals, the remaining strains are isolated from inpatient.

Among the 38 *E. coli* harboring carbapenemase genes, 15 distinct STs were identified. Notably, ST206 (36.84%, 14/38) was the predominant type, followed by ST1011 (10.52%, 4/38), ST533 (10.52%, 4/38), and ST167 (7.89%, 3/38) (Figure 3). ST206 remained the primary type in Henan province, suggesting a notable role of clonal dissemination in the propagation of carbapenemase-producing *E. coli* strains. Figure 3 provides further insight into the top 3 STs of *E. coli* in Zhejiang province. The results suggest the important role of non-clonal dissemination. Interestingly, ST155 was detected in both Henan and Shandong provinces. Regarding the 10 carbapenemase-producing *Klebsiella* isolates, five distinct STs were identified. ST2270 (60%, 6/10) was the most dominant, with a single isolate each of ST15, ST444, ST1040, and ST416. Another noteworthy observation was the close association between specific ST types and the presence of particular carbapenemase genes. Importantly, all ST types of *E. coli* harbored *bla_NDM−5_*. A similar phenomenon was observed in carbapenem-resistant *Klebsiella* isolates; ST2270, ST444, ST1040 and ST416 predominantly harbored *bla_NDM−1_*, while ST15 harbored *bla_NDM−5_* (Table 3).

**Table 3:** Sts of isolated carbapenemase-producing strains at different locations in China.

Location	Bacterial	No. of samples	ST(Number)	Type of samples
Zhejiang	*E. coli*	20	ST1011 (4)	Healthy
			ST533 (4)	Inpatient
			ST167 (3)	Inpatient
			ST6803 (2)	Inpatient
			ST48 (1)	Healthy
			others (6)	Inpatient
	*Klebsiella*	8	ST2270 (6)	Inpatient
			ST15 (1)	Inpatient
			ST444 (1)	Inpatient
	*Enterobacter hormaechei*	2	ST2657 (2)	Inpatient
Henan	*E. coli*	15	ST206 (14)	Inpatient
			ST155 (1)	Inpatient
	*Klebsiella*	2	ST1040 (1)	Inpatient
			ST416 (1)	Inpatient
	*Enterobacter hormaechei*	1	ST171 (1)	Inpatient
	*Citrobacter*	8	ST583 (3)	Inpatient
			ST352 (3)	Inpatient
			ST301 (1)	Inpatient
			ST118 (1)	Inpatient
Gansu	*Enterobacter hormaechei*	5	ST175 (4)	Inpatient
			ST171 (1)	Inpatient
	*Citrobacter portucalensis*	1	ST408 (1)	Inpatient
	*E. coli*	1	ST2520 (1)	Inpatient
Shandong	*E. coli*	2	ST155 (1)	Inpatient
			ST156(1)	Inpatient

A surprising observation concerned carbapenem-resistant *Citrobacter freundii*, ST118 collected from Henan province harbored two carbapenemase genes, *bla_NDM−1_* and *bla_IMP−4_*. Furthermore, ST175 and ST171 were observed among the eight carbapenemase-producing *E. hormaechei* in Gansu province, which harbored *bla_NDM−1_* gene. Conversely, an uncertain classification of *A. subteranean* isolate harbored *bla_IMP−4_*.

## 4. Discussion

CRE is a global crisis that is associated with high mortality rates and poses a severe threat to public health (27, 28). Comprehensive understanding of the epidemiology and symptoms of CRE are vital, especially in location active surveillance. This knowledge would help facilities determine control measures.

In this study, 9.6% of samples were characterized as CRE, with carriage rates of 4.26% from healthy individuals and 10.84% from hospitalized inpatients. These rates are higher than the rates of 6.6% and 8.5% in 2014 and 2019 studies from China, respectively (29, 30), but lower than the rates of 12.2% in Japan (31) and 37.9% in Iran (32). Previous studies among non-hospitalized human rectal swabs reported carriage rates of CRE of 0.6% in Vietnam (33), 0.4% in Spain (34), and 6.1% in India (35). However, the prevalence of CRE (10.84%) among hospitalized patients was a much lower than the 13% reported from Vietnam (36), and higher than the 2.9% rate in Spain (34). The increased prevalence may also be associated with failure to control CRE and broad-spectrum antibiotic use in hospitals. The previous studies were performed in hospitalized patients, acute rehabilitation units, long-term care hospitals, and acute-care hospitals. In the present study, we detected stool samples in healthy people and inpatients from four different provinces. It is pertinent to point out that a direct comparison of these studies should be done with caution, given the different settings, selective methods, and patients. Our results suggested that Henan province had a higher positive rate, which is consistent with previous study (37). We also observed that the rate of CRE was higher than the previous reported rate (38), because this study was conducted in ICUs.

The most commonly identified CRE was *E. coli* followed by *Klebsiella* and *Citrobacter*. A previous study reported different results (39). In another study, *Citrobacter* was the third most common isolates following *Klebsiella* and *E. coli*, similar to this study (40). *bla_NDM_* is the predominant resistance gene in *E. coli* strains. Other carbapenemase genes, such as *bla_IMP−4_*, were notably rare and detected only in a single isolate of *A. subterranean*. Simultaneously, one *C. freundii* strain, isolated from Henan province, harbored both *bla_NDM−1_* and *bla_IMP−4_*. These findings may indicate that *bla_NDM_* was the key carbapenemase gene responsible for mediating development of the carbapenem resistance phenotypes in China. Previous studies suggested *bla*_NDM−5_ was mostly located on a 46-kb IncX3 plasmids, which are increasingly associated with the dissemination of CRE (41, 42). Because of rapid and widespread of *bla_NDM_*, there is a pressing need to develop novel therapies to treat CRE producing NDM carbapenemase. However, current new antibiotics like ceftazidime-avibactam may not be suitable to remedy CRE in China (8).

MLST analysis showed that STs of CRE isolated in this study were genetically highly diverse. *E. coli* included 15 ST types, ST206 was the major type, followed by ST533 and ST1011. All strains harbored *bla_NDM−5_*. Other strain types were relatively rare and more sporadic. As for *Klebsiella*, ST2270, ST444, ST1040 and ST416 predominantly harbored *bla_NDM−1_*, while ST15 harbored *bla_NDM−5_*. A previous study suggested that ST11 of *K. pneumoniae* (mainly harboring *bla_kpc−2_*) was a major strain type in China (19). ST11, ST15, ST258, and ST512 are reportedly the main types in Europe and America (18). For *E. coli*, ST174 and ST648 that harbor *bla_NDM−5_* are prevalent in India (43). However, in Korea, ST101 harboring *bla_NDM−1_* is most prevalent (44). This variance may also be associated with the sample numbers and different regions in the different studies. Our findings showed the clonal and non-clonal transmission of *E. coli* harboring *bla_NDM_* in Henan and Zhejiang provinces, which reflects the high risk of specific clones disseminating.

There were several limitations in our study. These included the small sample size. The lack of discussion of the regional differences was the biggest flaw of this study. In addition, there were inconsistencies in collection of fecal samples from healthy and hospitalized individuals in provinces where fecal samples were collected. These inconsistencies may have biased the findings. Therefore, the true proportion of CRE in China was not able to be determined. Further studies should be performed.

In conclusion, a surveillance of healthy persons and hospitalized patients rectal carriage by CRE in four provinces in China was performed. We describe the prevalence of fecal colonization with CRE in these locations. The CRE positive rate in Henan province was highest, followed by Zhejiang province. ST206 of *E. coli* harboring *bla_NDM−5_*was most prevalent. The findings emphasized that early screening of CRE fecal colonization carriage in populations could clarify the prevalence of local CRE, and highlighted the importance for adopting measures to mitigate spread of CRE.

## Data Availability

The datasets presented in this study can be found in online repositories. The names of the repository/repositories and accession number(s) can be found below: [https://www.ncbi.nlm.nih.gov/bioproject / PRJNA1023209].
